# How Sugar Quality and Concentration Influence Oviposition Preference in *Drosophila Melanogaster*

**DOI:** 10.1007/s10886-025-01620-3

**Published:** 2025-06-20

**Authors:** Julio Otárola-Jiménez, Richard Spehr, Bill S. Hansson, Markus Knaden

**Affiliations:** 1 Department of Evolutionary Neuroethology, Max-Planck Institute for Chemical Ecology, 07745 Jena, Germany; 2 Chemistry School, University of Costa Rica, San Pedro, San José, 11501- 2060 Costa Rica

**Keywords:** Oviposition Behavior, *Drosophila*, Sugar Concentration, Larval Survival, Gustatory Cues

## Abstract

Female insects possess a complex chemosensory system that enables them to identify optimal oviposition substrates for their developing offspring. Both calorie-rich and protein-rich substrates are particularly attractive for laying eggs. We hypothesize that females detect concentrations of sweet compounds and preferentially select those concentrations that enhance larval survival. To test this, we investigated how female *Drosophila melanogaster* evaluate substrates with varying concentrations of sugars and amino acids, and whether these preferences correlate with larval survival. Using choice assays, we found that females preferentially deposited eggs on substrates containing 0.1 mol/L sucrose, while higher concentrations (2 mol/L) were consistently avoided—a response not attributable to differences in substrate hardness or prior feeding experience during mating. In contrast, when presented with monosaccharides, females favored intermediate concentrations: 0.5 mol/L for both glucose and fructose, with a marked overall preference for fructose even when combined with an amino acid mixture. Moreover, substrates aligning with the females’ oviposition choices often supported best survival of larvae. These findings indicate that female flies assess not only the concentration but might also consider the chemical nature of sweet compounds when making oviposition decisions and they underscore a critical link between maternal site selection and larval development.

**Supplementary Information:**

The online version contains supplementary material available at 10.1007/s10886-025-01620-3.

## Introduction

In the animal kingdom, maternal decisions can significantly influence offspring survival and fitness. In insects, the egg-laying decision of the male often dictates the first and most critical environment encountered by the developing young. Consequently, gravid females must integrate diverse environmental cues—ranging from nutrient availability to chemical toxicity—when selecting optimal oviposition substrates. This decision-making process is shaped by a sophisticated chemosensory system that allows females to evaluate the suitability of potential sites based on external signals and internal physiological state(Stocker 1994; Tom et al. 2022; Vosshall and Stocker 2007; Wu et al. 2020).

Over the past decades, research has made significant progress in uncovering how insects perceive and evaluate food-derived nutrients (Hendriksma and Shafir 2016; Leach and Drummond 2018; Ruedenauer et al. 2023). In particular, the molecular mechanisms underlying the detection of sugars (Frank et al. 2024; Gomes et al. 2024; Ma et al. 2024; Miyamoto et al. 2013), bitter compounds (Ahn and Amrein 2023; Kim et al. 2016), amino acids (Aryal et al. 2022; Park and Carlson 2018), and other substances (Chen et al. 2010; Jiang et al. 2020) have been increasingly elucidated (for reviews, see Y.-C. D. Chen and Dahanukar 2020; Liman et al. 2014; Montell 2009; Yarmolinsky et al. 2009).

In *Drosophila*, taste is mediated by sensory bristles located on the proboscis, legs, wings, and genitalia in adults (Montell 2009; Stocker 1994), as well as by larval sensory structures such as the dorsal, terminal, and ventral organs (Apostolopoulou et al. 2015). Two major families of receptors underlie this sensory capacity: gustatory receptors (Grs), comprising 68 members (Clyne et al. 2000; Dunipace et al. 2001; Robertson et al. 2003; Scott et al. 2001), and ionotropic receptors (Irs) (Benton et al. 2009; Stewart et al. 2015). Unlike mammals, which rely on a single heterodimeric pair of G-protein-coupled receptors to detect sugars, *Drosophila* flies employ a combinatorial approach using at least 8 Grs (Dahanukar et al. 2007; Liman et al. 2014; Scott 2005; Slone et al. 2007). For example, sucrose and glucose detection in adults involves Gr5a, Gr64a, and Gr64f (Jiao et al. 2007, 2008; Liman et al. 2014), while Gr43a functions as a fructose receptor in adults (Miyamoto et al. 2012), and seems to be the primary sweet receptor in larvae (Maier et al. 2021; Mishra et al. 2013).

When choosing oviposition sites, female *Drosophila* must balance their own nutritional needs with the predicted survival and development of their offspring. Evidence suggests that sexual dimorphism in sensory structures—specifically, the presence of additional bristles on the female genitalia—enhances the female’s ability to evaluate substrate quality (Rice 1977; Scott et al. 2001; Taylor 1989). Protein-rich substrates, which support egg production, are highly attractive (Lihoreau et al. 2016; Vargas et al. 2010). Energy-providing sugars also play a role in oviposition preference, although the nature of this influence is less well understood (Chen et al. 2022; Durkin et al. 2021; Lihoreau et al. 2016; Liu et al. 2017; Schwartz et al. 2012; Vijayan et al. 2022, 2023; Wang et al. 2022). These nutrients not only influence maternal behavior but also affect larval traits such as body size, fat storage, and lifespan (Millington et al. 2022; Pasco and Léopold 2012; Reis 2016).

Sugars such as sucrose, fructose, and glucose are abundant in fruits, but their concentrations vary depending on fruit type (Moroz 2024; U.S. Department of Agriculture 2025) and ripeness (Mahmood et al. 2012; Solanky et al. 2015). According to international food composition databases, sugar concentrations in fruit range from 3.8 to 13 g/100 g, equivalent to a range of 0.10–0.67 mol/L, assuming a fruit density of 0.933 g/mL (Liu et al. 2025).

Behavioral studies have demonstrated that *Drosophila* can detect and discriminate between different sweet compounds, responding to both the type and concentration of sugars (Fujita and Tanimura 2011; Marella et al. 2006; Wang et al. 2004). In oviposition context, both *D. melanogaster* and *D. suzukii* tend to prefer sweet substrates, yet *D. suzukii* displays a weaker preference for high sugar concentrations (Cavey et al. 2023; Wang et al. 2022). Intriguingly, other studies have reported avoidance of sugary oviposition substrates altogether (Chen et al. 2022; Yang et al. 2008). Such conflicting results may reflect differences in experimental design or in the range of concentration tested—ranging from 0 to 0.6 mol/L (Cavey et al. 2023; Chen et al. 2022; Durkin et al. 2021; Wang et al. 2022).

These findings suggest that sugars are not a simple cue, but a complex and context-dependent signal. While sugars can serve as a proxy for energy-rich environments, excessively high concentrations may signal microbial spoilage (Kienzle and Rohlfs 2021) or osmotic stress (Pierce et al. 1999), which could compromise larval survival. Indeed, *Drosophila* larvae are capable of evaluating both the nutritional value and palatability of sugars, showing peak attraction and feeding behavior at intermediate concentrations of sucrose and fructose, while higher concentrations may be avoided (Schipanski et al. 2008; Ugrankar et al. 2018).

In this light, maternal oviposition behavior might reflect a finely tuned assessment of substrate quality, not merely for the adult female’s benefit but for the long-term success of her progeny. Is it possible that female flies “know” what’s best for their offspring—discerning between sugar types and rejecting concentrations that could be harmful to larval development? Does this behavioral flexibility represent an evolved strategy that balances attraction to nutritional cues with avoidance of potentially toxic extremes?

Despite growing insight into sugar perception and preference, it remains unclear whether *D. melanogaster* females exhibit distinct concentration thresholds during oviposition decisions or whether they differentially prioritize sugar identity when evaluating potential egg-laying sites. In essence, does the mother truly *know better*?

To address this question, we investigated how sugar type and concentration influence oviposition site selection in *D. melanogaster*. Using two- and four-choice assays, we tested whether females can discriminate among substrates based on sugar quality and quantity, and whether their preferences align with ecologically relevant sugar concentrations.

## Results

**Fig. 1:**
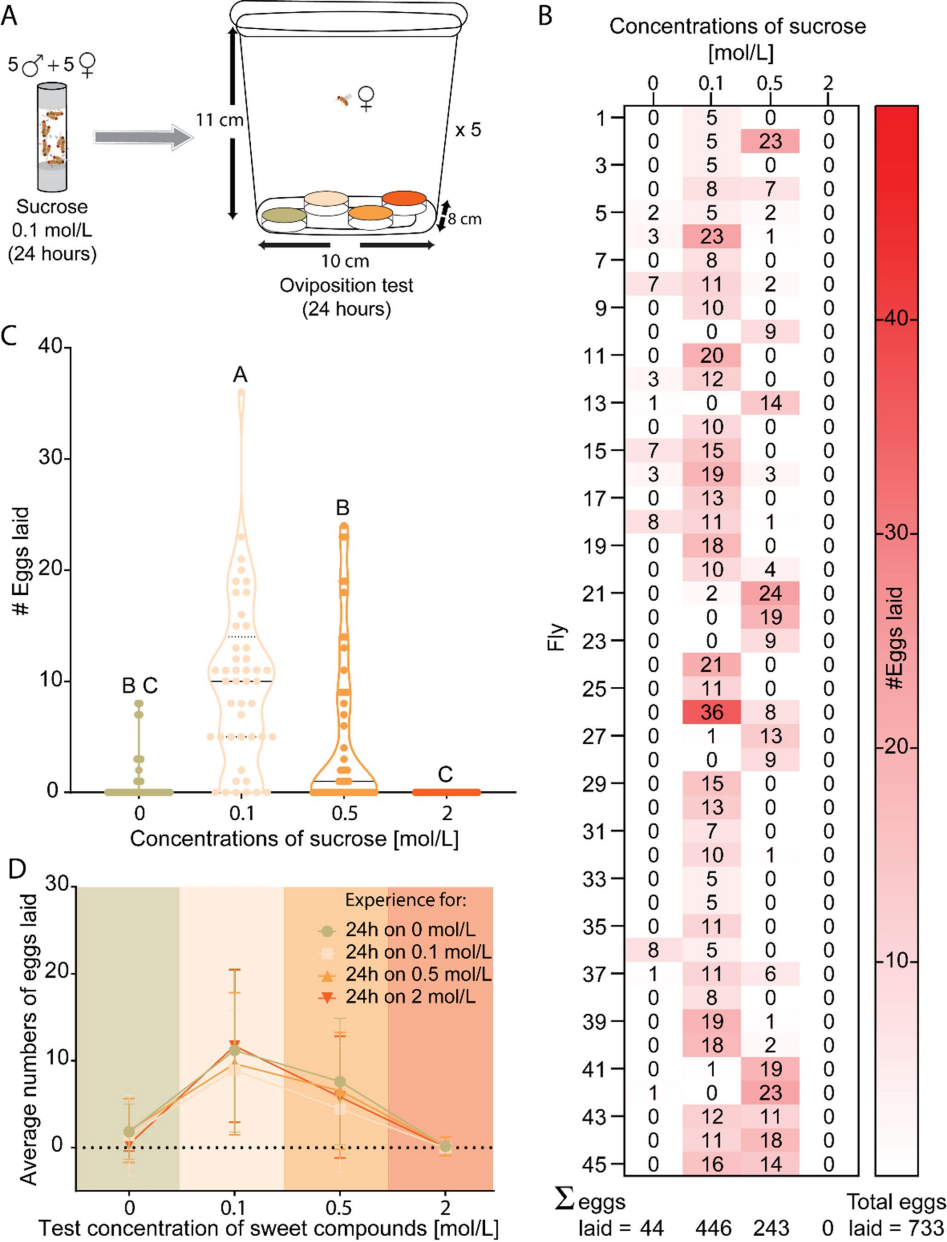
Females prefer to oviposit on 0.1 mol/L sucrose. **(A)** Schematic of the paradigm used to test oviposition preference of individual *D. melanogaster* females. **(B)** Heat map showing the number of eggs each female laid over 24 h on neutral substrate (0 mol/L, 0.3% w/v agar) and on three different sucrose concentrations of sucrose (*n* = 45). **(C)** Comparison of the number of eggs laid during the oviposition test on different sucrose concentrations. A *Friedman test* followed by *Dunn`s test* for multiple comparison was used to test for significance (α = 0.5, *n* = 45). Significant differences (*P* < 0.05) among concentrations are shown with the Compact Letter Display (CLD) system on the top of each violin plot. For the hardness of the substrates used see Figure S1D. **(D)** Oviposition preference of females that had prior experience on different sucrose concentrations before the test. Symbols depict the pre-treatments on different sucrose concentration, and colored columns depict the test concentration. Not all the data exhibited a Gaussian distribution (*Shapiro-Wilk test*). Therefore, a *mixed-effects model* was used to assess the effect of repeated measures of the prior experience on oviposition preference (*n* = 43 for each treatment, α = 0.5, total number of eggs in the pre-treatments on 0, 0.1, 0.2, and 2 mol/L were 893, 723, 788, and 772, respectively. Fixed effects: substrate concentration, pre-treatment concentration, and substrate concentration x pre-treatment concentration)

We followed a protocol similar to the one described in our previous study (Otárola-Jiménez et al. 2024). Five virgin females and five males were placed in a vial for 24 h with an absorbent plug soaked in 5 mL of 0.1 mol/L sucrose solution, which prevented oviposition but avoided starvation. Afterwards, a single gravid female was transferred to a four-choice assay to assess its oviposition preferences among different substrates (Fig. 1A).

*Female Flies Prefer to Oviposit at a Specific Sucrose Concentration*. First, we examined oviposition preferences between a neutral substrate (0.3% w/v agarose) and three sucrose concentrations (0.1, 0.5, and 2 mol/L). Our decision to work with four substrate is based on the maximum number of Petri dishes that we can put in the oviposition chamber, and the range of concentrations was selected based on the natural concentration of sugars that can be found in ripe fruits (U.S. Department of Agriculture 2025) and that have been used in behavioral experiments related with oviposition (Cavey et al. 2023; Durkin et al. 2021; Wang et al. 2022). The selection of 2 mol/L was made based on the experiments where sugars are used as rewards in learning experiments (Kim et al. 2007; Schipanski et al. 2008).

In this four-choice assay, females did not lay any egg on the 2 mol/L substrate (Fig. 1B) and deposited only a small number on the neutral substrate (44 out of 733, i.e. less than 6%). However, the female flies laid more eggs on 0.1 mol/L substrates (446 out of 733, i.e. more than 60%), and then on 0.5 mol/L substrate (243 out of 733, i.e. 33%) (Fig. 1C). These results suggest that the relationship between sucrose concentration and oviposition preference is not linear and that female flies actively evaluate sucrose concentration, finding 0.1 mol/L particularly more attractive.

However, it remains unclear why females prefer this specific concentration. Do they taste and innately select the optimal concentration, or might other factors influence their choice, such as substrate hardness, prior exposure to 0.1 mol/L sucrose during mating, or even the presence of multiple choices in the assay? To address these questions, we investigated how each of these factors might affect the oviposition decisions of gravid females.

In all experiments, the positions of the four substrates were randomized to prevent any bias caused by neighboring concentrations. Next, we varied the number of choices in the oviposition test. Instead of a four-choice assay, we conducted a two-choice assay (Figure S1A). When female flies were presented with the choice between a neutral substrate (0.3% w/v agarose) and 0.1 mol/L sucrose, they laid significantly more eggs on the sweet substrate (Figure S1B). However, when given a choice between a neutral substrate and 0.5 mol/L sucrose, there was no significant preference for either option (Figure S1C). Interestingly, when female flies were presented with the choice between 0.1 mol/L and 0.5 mol/L, they laid significantly more eggs on 0.1 mol/L (Figure S1D). Moreover, there was no difference in the total number of eggs laid in both two-choice assays that included neutral substrates (Figures S1B, S1C). However, when two sweet substrates (0.1 and 0.5 mol/L) were used in the two-choice assay the total number of eggs laid decreased (Figure S1D). Overall, these results support that the relationship between sucrose concentration and oviposition preference is not linear, and that higher concentrations, such as 0.5 mol/L, may become less attractive. These findings agree with our four-choice assay results, indicating that the oviposition preference for 0.1 mol/L sucrose remains consistent regardless of whether a two- or four-choice paradigm is used.

As female *Drosophila melanogaster* flies typically prefer soft substrates for oviposition (Yapici 2020; Zhang et al. 2020), we measured the hardness of the neutral substrate and of each sucrose concentration (Figure S1E). We used a force gauge with a small indenter designed to pierce the substrate. The 2 mol/L sucrose substrate was significantly harder than the other substrates, which likely contributed to the absence of eggs laid on it. Furthermore, we observed that a sticky layer was formed on the 2 mol/L substrate within two hours after preparation, potentially making it even less attractive for oviposition. In contrast, the other three substrates did not differ significantly in hardness, indicating that hardness alone does not affect the oviposition preference towards the 0.1 mol/L sucrose substrate.

Previous research has shown that prior experience can alter insect oviposition preferences (Gámez and León 2018; Hu et al. 2018; Nataraj et al. 2021; Otárola-Jiménez et al. 2024; Proffit et al. 2015; Xie et al. 2021). We therefore asked whether feeding experience during mating could enhance the oviposition preference for 0.1 mol/L sucrose. Following the protocol in Fig. 1A, females were mated in the presence of 0, 0.1, 0.5, or 2 mol/L sucrose solution before their oviposition preferences were tested (Fig. 1D). In all treatments, the flies consistently preferred to lay eggs on 0.1 mol/L sucrose (*Mixed-effects model [REML]*, fixed effect of substrate concentration: F(1.464, 327.9) = 115.4, P = < 0.001, Fig. 1D), indicating that feeding experience during mating did not influence oviposition preference (*Mixed-effects model [REML]*, fixed effect of pre-treatment concentration: F(3, 672) = 1.987, *P* > 0.05, Fig. 1D).

Overall, our results demonstrate that neither substrate hardness, feeding experience, nor the arrangement or number of available choices influence the oviposition preference, which remains for 0.1 mol/L sucrose. We propose that females favor this concentration because it provides optimal sweetness conditions for both the females and, potentially, their larvae—a hypothesis we later tested.

There is evidence that individual gustatory receptors can respond to multiple sugars (Fujii et al. 2015; Jiao et al. 2007) and that their functional roles may vary depending on their anatomical site of expression (Kohatsu et al. 2022; Ling et al. 2014; Thoma et al. 2016). This raised the question of whether oviposition behavior in response to varying concentrations of fructose or glucose would differ from the patterns we previously observed with sucrose.

**Fig. 2:**
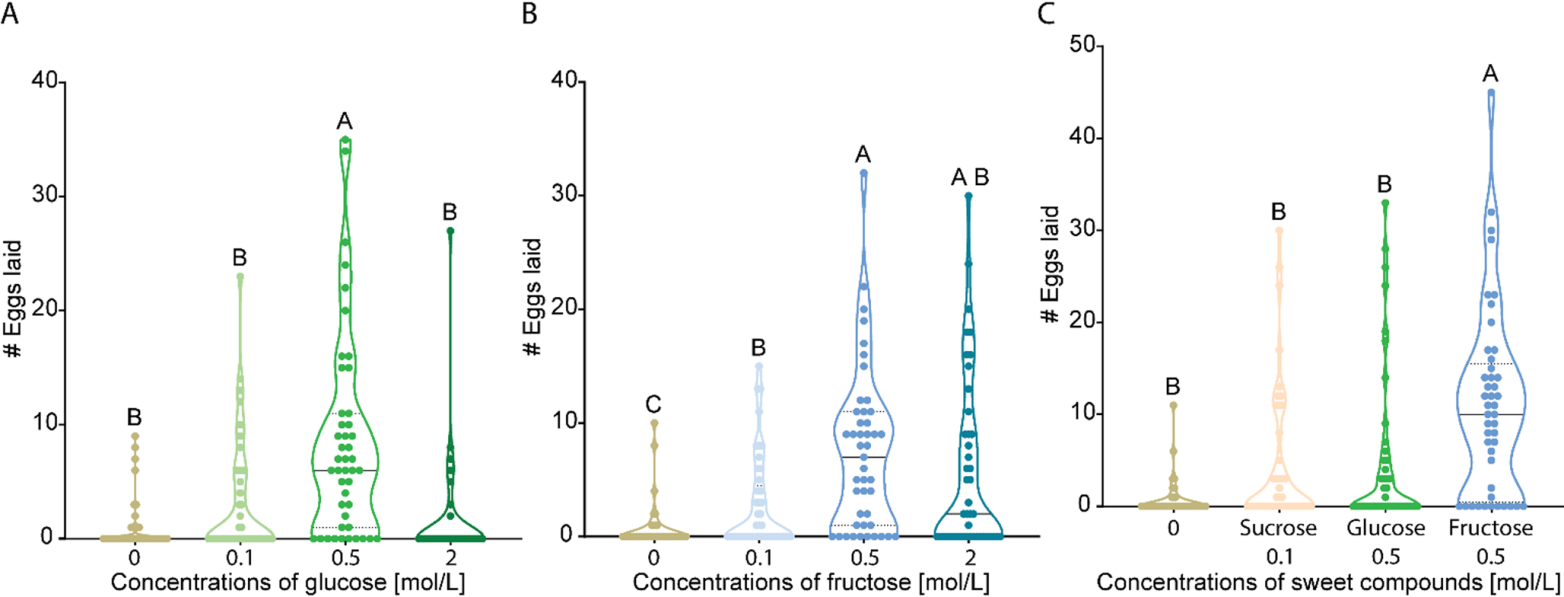
Females *D. melanogaster* show distinct oviposition preferences based on both chemical identity and concentration. **(A)** Comparison of the number of eggs laid during the oviposition tests with various glucose concentrations. See Figure S2A for the total number of eggs laid by each female and Figure S3B for substrate hardness. **B)** Comparison of the number of eggs laid during the oviposition test with various fructose concentrations. See Figure S2B for the total number of eggs laid by each female and Figure S3C for substrate hardness. C) Comparison of the number of eggs laid during the oviposition test on neutral substrate (0 mol/L), 0.1 mol/L sucrose, 0.5 mol/L glucose, and 0.5 mol/L fructose. See Figure S2C for the total number of eggs laid by each female and Figure S3D for substrate hardness **(A-C)** *Friedman test* followed by *Dunn`s test* for multiple comparison test was used (α = 0.5, *n* = 45). Significant differences (*P* < 0.05) among concentrations are shown with the Compact Letter Display (CLD) system on the top of each violin plot

*Female Flies Prefer to Oviposit at Intermediate Concentrations when Detecting Glucose or Fructose*. It has been shown that *Drosophila* females prefer fructose or glucose substrates over neutral substrates (Wang et al. 2022). However, it is unclear if they show any oviposition preference for specific concentrations of these sugars, as we found for 0.1 mol/L sucrose. To explore this, we again conducted a four-choice assay featuring a neutral substrate and three substrates featuring different glucose concentrations. Under these conditions, females deposited significantly more eggs on 0.5 mol/L glucose (Fig. 2A), accounting for over 61% of the total number of eggs laid (Figure S2A). These results indicate that, in contrast to sucrose, females prefer higher glucose concentrations for oviposition.

When fructose was tested, we observed a marked preference for higher fructose concentrations overall, though there was no significant difference in the number of eggs laid on 0.5 mol/L versus 2 mol/L (Fig. 2B). Specifically, females deposited 45% of their eggs at 0.5 mol/L, 35% at 2 mol/L, and only 16% and 4% at 0.1 mol/L and 0 mol/L, respectively (Figure S2B). These findings indicate that females generally favor higher fructose concentrations compared to sucrose. However, this preference appears to have an upper threshold, as females showed a significant preference for 2 mol/L over 4 mol/L fructose (Figure S3A). Taken together, these data suggest that 0.5 mol/L is the preferred fructose concentration for oviposition.

To examine whether substrate hardness influenced the flies’ preferences, we measured the hardness of the glucose (Figure S3B) and fructose (Figure S3C) substrates in the same manner as for sucrose. We found no significant differences in hardness among the most preferred concentrations, nor between these concentrations and the neutral substrate. Thus, hardness does not appear to guide the oviposition decisions of female flies when sensing glucose or fructose.

Finally, as our results show that flies prefer intermediate concentrations of glucose and fructose for oviposition, we also wondered whether females would display any sugar-specific preference during oviposition when exposed to the most favored concentrations of sucrose, glucose, and fructose.

*Female Flies Prefer to Lay Eggs on Fructose-Containing Substrates*. Given that female flies prefer to oviposit on 0.1 mol/L sucrose, 0.5 mol/L glucose, and 0.5 mol/L fructose, we asked whether females would prefer one sweet compound over the others during oviposition. To investigate this, we performed a four-choice assay using the most preferred concentrations of each sweet compound alongside a neutral substrate (Fig. 2C). The results show that females significantly prefer to oviposit on 0.5 mol/L fructose, depositing more than 52% of their eggs on this substrate (Figure S2C). This finding might indicate that, beyond detecting only concentration differences, females might also assess the specific type of sugar when deciding where to oviposit. However, whether flies really can do so needs further testing (see for different results in a aversive training assay Masek and Scott 2010).

**Fig. 3:**
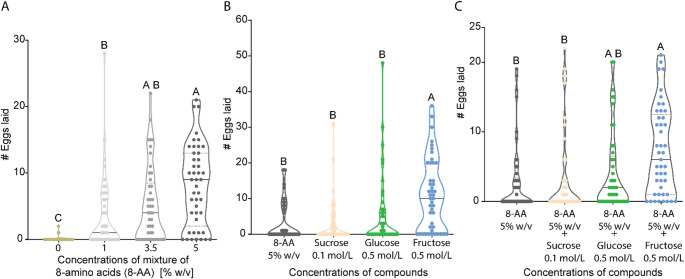
Females prefer to oviposit on substrates with 0.5 mol/L fructose. **(A)** Comparison of the number of eggs laid during the oviposition test using various amino acid concentrations. See Figure S4A for the total number of eggs laid by each female and Figure S4D for substrate hardness. **(B)** Comparison of the number of eggs laid during the oviposition test on 5% w/v amino acids mixture, 0.1 mol/L sucrose, 0.5 mol/L glucose, and 0.5 mol/L fructose. See Figure S4B for the total number of eggs laid by each female and Figure S4E for substrate hardness. **(C)** Comparison of the number of eggs laid during the oviposition test on 5% w/v mixture of amino acids (8-AA), 5% w/v 8-AA + 0.1 mol/L sucrose, 5% w/v 8-AA + 0.5 mol/L glucose, and 5% w/v + 0.5 mol/L fructose. See Figure S4C for the total number of eggs laid by each female and Figure S4F for substrate hardness. **(A-C)** *Friedman test* followed by *Dunn`s test* for multiple comparison test was performed (α = 0.5, *n* = 45). Significant differences (*P* < 0.05) among concentrations are shown with the Compact Letter Display (CLD) system on the top of each violin plot

One critical factor during searching for a suitable substrate is protein availability, as females can metabolize protein and use its amino acids for egg production (Alves et al. 2022; Lihoreau et al. 2016; Vargas et al. 2010). We, therefore, examined whether females also display a threshold concentration during oviposition on different amino acid concentrations by using a commercial mixture of eight essential amino acids (8-AA; see Table S1 for composition). Our results show that the higher the amino acid concentration, the higher the number of eggs laid (Fig. 3A, S4A). However, we were unable to determine the most preferred concentration, as the amino acid mixture became insoluble at 7% w/v, leading to agglomeration on the substrate.

Next, we evaluated oviposition preference on 5% w/v 8-AA substrates alongside the most preferred sweet compound concentrations. Although the presence of eight essential amino acids can enhance egg production (Alves et al. 2022; Grandison et al. 2009), females still preferred the substrate that contained their most preferred sugar, i.e., 0.5 mol/L fructose (Fig. 3B, S4B). This finding is in conflict with a previous study, which demonstrated that females consistently favor amino acid over sucrose in a binary feeding assay (Ganguly et al. 2017). However, the results of this study are based on feeding assays.

Previous studies in other insects suggest a synergistic interaction between sugars and amino acids when presented in mixtures (Alm et al. 1990; Ganguly et al. 2017; Wada et al. 2001). Therefore, we combined the most preferred sweet compound concentration with 5% w/v 8-AA (Fig. 3C) and tested the oviposition preference. Female flies showed similar preferences as when the sugars were tested without the amino acids, preferring the substrate of 0.5 mol/L fructose with more than 40% of all the eggs laid (Figure S4C). Moreover, hardness measurements showed that substrate consistency did not influence this decision, as no significant differences in hardness were detected among the four substrates (Figure S4F).

So far, we have demonstrated that female flies exhibit a specific oviposition preference for certain concentrations, depending on the type of sugar. Notably, they strongly prefer fructose, even in the presence of amino acids. We next wondered what the development or survival of Drosophila larvae would be at those concentrations where female flies preferred to lay more eggs.

*Larvae Exhibit Higher Survival Rates at Concentrations where Female Flies Prefer to Oviposit*. It is known that protein (yeast) and sugar are essential to larvae for an optimal development (Schwarz et al. 2014). Of course, without a proper balance of these essential nutrients larvae cannot survive. We evaluated the larval survival on substrates containing the different concentrations of sugars, amino acids, and combinations of both. Neutral substrates were used as a control to determine how the quality and quantity of each chemical compound affect the larval development. For each concentration, we prepared ten Petri dishes (35 × 10 mm), each containing ten eggs, resulting in a total of 100 eggs per concentration. The number of hatched and surviving larvae was recorded on a daily base, and each larva that did not hatch or died on a given day was counted as an event. All larvae died before or directly after reaching the second instar.

**Fig. 4:**
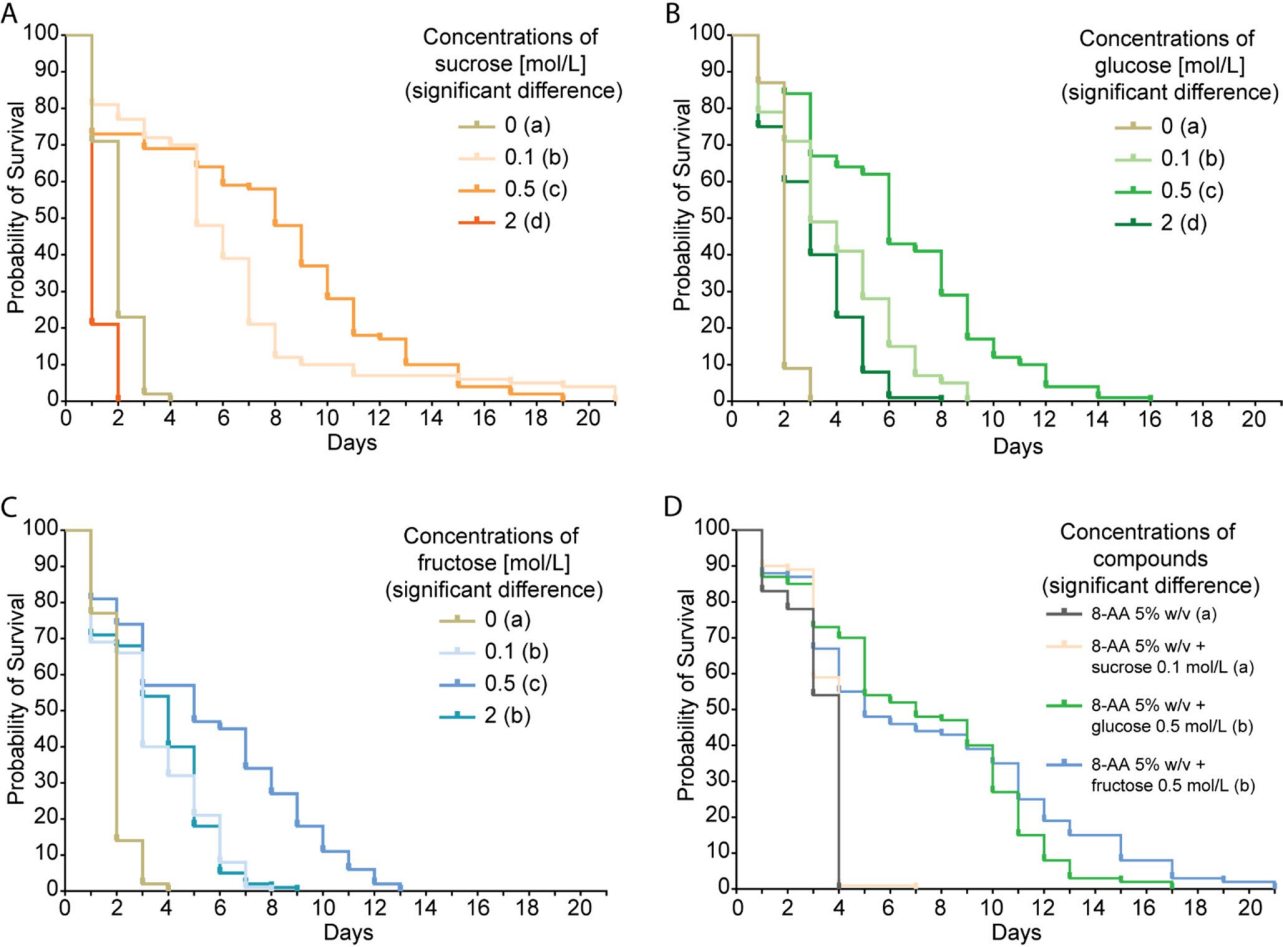
Larvae survive longer on those substrates were females laid more eggs. **(A)** Kaplan-Meier survival curve of *Drosophila* larvae on different sucrose concentrations. **(B)** Kaplan-Meier survival curve of *Drosophila* larvae on different glucose concentrations. **(C)** Kaplan-Meier survival curve of *Drosophila* larvae on different fructose concentrations. **(D)** Kaplan-Meier survival curve of *Drosophila* larvae on 5% w/v mixture of amino acids (8-AA), 5% w/v 8-AA + 0.1 mol/L sucrose, 5% w/v 8-AA + 0.5 mol/L glucose, and 5% w/v + 0.5 mol/L fructose. **(A-D)** *Log-rank (Mantel-cox) test* with pairwise comparison and *Bonferroni’s’ test* for multiple comparison test were performed (α = 0.5, # of larvae per treatment = 100). Significant differences (*P* < 0.05), are shown with the Compact Letter Display (CLD) system in lower case on the right side of the legends in each graph

Once all the events of the larvae were recorded, survival rates were plotted as Kaplan-Meier survival curves for the different concentrations of compounds (Fig. 4). To asses concentration-dependent differences in survival, we performed a Log-rank (Mantel-cox) test with pairwise comparison, including a post hoc test for multiple comparisons. Additionally, we examined the larval median survival time (which is the time point when 50% of the population had died) as a metric to compare survival durations across treatments.

For sucrose, all survival curves differed significantly (Fig. 4A). The highest median survival (8 days) was observed at 0.5 mol/L, whereas the lowest (1 day) was observed at 2 mol/L. These findings only partially align with female oviposition preferences, as females showed a marked oviposition preference for 0.1 mol/L sucrose (Fig. 1C). However, the substrate that received no eggs at all (2 mol/L sucrose) also had the lowest median survival.

For both glucose and fructose, all survival curves differed significantly (Fig. 4B and C). Notably, these results aligned closely with female oviposition preferences: the substrates most preferred by ovipositing females (0.5 mol/L glucose or fructose; Fig. 2A) were also those on which larvae exhibited the longest median survival duration.

Similarly, when larvae were reared on combinations of amino acids and sugars (Fig. 4D), the longest survival durations were observed on the same substrates (amino acids combined with glucose or fructose) that had previously elicited the strongest oviposition preferences from females (Fig. 3C).

To analyze if there is a correlation between the oviposition preference and the larval survival, we performed a Kendall´s rank correlation across all conditions, using the mean of the eggs laid and the median survival of larvae of the different concentrations of compounds. We found a strong positive correlation (Figure S5, τ = 0.738, *z* = 3.84, *p* < 0.001) suggesting that female oviposition preference aligns with larval performance. However, it is important to mention that regardless of the compounds tested, none of the larvae finally pupated, highlighting that for successful development crucial compounds were missing in all conditions.

## Discussion

Gravid *Drosophila* females use multiple sensory cues, including gustation, to assess oviposition sites, often favoring protein- and sugar-rich substrates that enhance reproductive success (Durkin et al. 2021; Lihoreau et al. 2016; Liu et al. 2017; Vargas et al. 2010; Vijayan et al. 2023; Wang et al. 2022). While several studies report a preference for sweet over neutral substrates (Cavey et al. 2023; Durkin et al. 2021; Wang et al. 2022), others show the opposite trend, with females preferring neutral media (Chen et al. 2022; Vijayan et al. 2022; Yang et al. 2008). One likely explanation for these conflicting results is the size of the oviposition arena. Studies reporting a preference for neutral substrates often used smaller (e.g., 16 × 10 × 6 mm; Gou et al. 2016), whereas those reporting a preference for sweet substrates used larger arenas (e.g., 24.5 × 24.5 × 24.5 cm; Wang et al. 2022). This suggests that spatial constraints may influence oviposition decisions. As Schwartz et al. (2012) showed, larval foraging costs increase in large arenas. When movement is restricted and substrates are spatially separated—as in our design—females may lay eggs directly on sweet substrates to minimize foraging and enhance larval survival. In contrast, in smaller arenas where larvae can freely access all substrates, precise placement by the mother becomes less critical, possibly leading to more indifferent oviposition behavior.

In our study we found that female flies prefer to lay eggs on agar containing sugar and that this preference was concentration dependent. When tested e.g. with sucrose, flies most preferred a concentration of 0.1 mol/L. This concentration of sucrose is widely used in experiments using the proboscis extension response (PER) and in oviposition experiments (Dahanukar et al. 2007; Fujita and Tanimura 2011; Joseph et al. 2017; Kim et al. 2007; Mermer et al. 2022; Wang et al. 2022), although its prevalence in the literature lacks clear justification. Notably, in our study higher concentrations, particularly those exceeding 1 mol/L, were avoided, despite being frequently used as unconditioned stimuli in feeding and learning assays (de Araujo 2011; Fujita and Tanimura 2011; Kim et al. 2007). Interestingly, high sugar concentrations may have physiological costs. Studies in the context of dietary restriction and aging have shown that fecundity declines when flies are fed sucrose concentration above 0.3 mol/L (Bass et al. 2007; Lushchak et al. 2014), potentially contributing to the reduced attractiveness of 0.5 mol/L sucrose in the oviposition context. These findings collectively support the notion that females assess sucrose differentially for feeding and oviposition, exhibiting an innate preference for moderate concentrations, specifically for 0.1 mol/l.

While our study did not evaluate concentrations below 0.1 mol/L, prior work by Wang et al. 2022 showed that in two-choice assays, *D. melanogaster* females consistently preferred to oviposit on 0.1 mol/L sucrose over lower concentrations (0, 0.01, 0.03, and 0.06 mol/L), further supporting our conclusion that this concentration represents a preferred threshold for oviposition. When testing the flies’ oviposition preference for the concentration of different sugars like glucose and fructose, we again found an optimum concentration, that this time was at 0.5 mol/L, but significantly preferred fructose in a direct comparison (Fig. 2C).

*Drosophila melanogaster* can detect a range of sweet compounds, including disaccharides like sucrose (Jiao et al. 2007; Joseph et al. 2017; Ma et al. 2024) and monosaccharides such as glucose and fructose (Fujii et al. 2015; Miyamoto et al. 2012, 2013; Miyamoto and Amrein 2014; Slone et al. 2007). Although glucose and fructose share the same molecular formula (C₆H₁₂O₆), their structural differences—hemiacetal vs. hemiketal groups—may influence detection. When all three sugars were presented simultaneously in a four-choice assay, sucrose consistently emerged as the least preferred and fructose the most preferred oviposition substrate (Figures S2C, S4B, and S4C). This pattern is consistent with previous no-choice studies showing that flies lay fewer eggs on sucrose-containing media (Bass et al. 2007; Lushchak et al. 2014). These findings may also help explain the relatively low number of eggs laid in our two-choice assay between two concentrations of sucrose (Figure S1D). In addition to reduced fecundity, sucrose consumption has been associated with decreased fertility in *Drosophila* (Brookheart et al. 2017), while fructose and glucose consumption extended longevity and fecundity was highest (Lushchak et al. 2014). Additionally, the preference for higher amounts of fructose and glucose (Figs. 2B and C) may be due to the fact that overripe fruits—the natural oviposition site of *Drosophila melanogaster*—contain higher levels of these monosaccharides than of sucrose (Mahmood et al. 2012; Mikulic-Petkovsek et al. 2023; Simkova et al. 2024; Widdowson and McCance 1935).

We also found that amino acid induced oviposition in a concentration-dependent manner, with flies preferring the highest concentrations tested (Fig. 3A). Amino acids, which are essential for development and reproduction across all life stages (Ma et al. 2022; Piper et al. 2017), appear to play a significant role in substrate evaluation. *Drosophila* can detect amino acids via gustatory and ionotropic receptors (Alves et al. 2022), and responses to amino acids in feeding assays are concentration-dependent (Aryal et al. 2022; Ganguly et al. 2017; Park and Carlson 2018).

In addition to oviposition preference, we also tested, how *Drosophila* larvae would develop on the different concentrations of sugars and on combinations of sugars and amino acids (Fig. 4). Calorie-rich substrates are essential for larval growth and survival (Schwarz et al. 2014). In behavioral assays, larvae have been shown to prefer substrates containing glucose, fructose, or sucrose over neutral media, with the strongest preference observed for 0.5 mol/L fructose and 0.1 mol/L sucrose (Mishra et al. 2013; Schipanski et al. 2008). Although, probably due to the lack of crucial nutrients, in our study none of the tested larvae survived until pupation, we found significant differences in survival durations between the test situations. Interestingly, the larvae did not perform best on the highest concentrations. It has been shown that high sugar concentration promotes obesity, whereas low concentration induces oxidative stress (Rovenko et al. 2015). At the same time osmotic pressure can negatively affect negatively larval physiology and sensory responses (Apostolopoulou et al. 2015; Zwaan et al. 1991). In previous studies assessing larval survival on sugar-containing substrates larvae survived until pupation. However those studies typically used third-instar (L3) larvae that were reared on nutritionally complete media during earlier stages of development (Rohwedder et al. 2012; Schipanski et al. 2008; Ugrankar et al. 2018). In contrast, our study assessed survival beginning at the L1 stage, immediately after egg laying, in order to evaluate how specific sweet compounds and their concentrations impact development from the beginning. In addition, this design allowed us to directly relate larval survival to oviposition preference. Interestingly, we found that larval survival duration was positively correlated with the flies’ oviposition preferences (Figure S5). Although the lack of pupation of any larvae in the assays show that crucial nutrients for development were missing, the correlation between female preference and larval survival duration might still suggest that the females discriminate the different substances and (although none of the substrates is good enough for survival) at least are able to identify the best one.

However, our findings contrast with a study by Liu et al. (2017), which reported an uncoupling of oviposition preference and larval survival when flies were offered sucrose substrates of varying concentrations. In their study, higher sucrose concentrations were associated with decreased oviposition, despite offering favorable conditions for larval development. The discrepancy between our findings and those of Liu et al. may be explained, in part, by differences in experimental design and the effect of arena size. Interestingly, they also found that when oviposition and larval survival were tested in the context of microbial presence, there was a clear coupling between maternal preference and offspring performance. Apparently, under certain biologically relevant conditions, female *Drosophila* can integrate multiple cues to select oviposition sites that are beneficial for their progeny.

## Methods and Materials

*Fly Stock*. This study used wild-type flies that were acquired from the National Drosophila Species Stock Centre (NDSSC; http://blogs.cornell.edu/drosophila/): *D. melanogaster* (Hansson’s Lab). All the flies were reared on yeast-cornmeal-agar medium (standard food), kept at 25 °C with 70% humidity. A 12 h:12 h light: dark cycle was established. Flies were collected and sexed during the first 3 h after eclosion, using ice as an anesthetic. Groups of 5 female and 5 male flies were kept separately in small vials with standard food until the start of the experiments. Flies were treated under the same conditions for all experiments. The *D. melanogaster* (wild type) stock used in this study and associated references are listed in the key resources table.

For 1 L of the standard food, the following ingredients were used: beet syrup (118 g), brewer’s yeast (11 g), agar powder (4.1 g), hot water (540 mL), corn grits (95 g), propionic acid (2.4 mL), nipagin 30% (3.3 mL), and cold water (378 mL).

### Oviposition Substrates

*Oviposition Sugar-Rich Substrates*. Glucose, fructose, or sucrose in 0.3% (w/v) agarose were used as oviposition substrates. For each experiment, a freshly prepared substrate was used, ensuring the sugar was mixed into the agarose to maintain a consistent softness.

To prepare 1000 mL of plain (neutral) substrate, 3 g of agarose (Agar-Agar, Kobe I, Art. No. 5210.2, Carl Roth GmbH) was dissolved in 1000 mL of distilled water and brought to boiling, resulting in a 0.3% (w/v) agarose solution. Then, 5 mL of this neutral agarose solution was added into 35 × 10 mm Petri dishes (Ref. No. 734–2314, VMR).

For the sucrose solutions, 3.42 g, 17.11 g, or 68.46 g of D(+)-sucrose (> 99.5% p.a., Carl Roth GmbH) was added to 100 mL of the hot (~ 70 °C) 0.3% (w/v) agarose solution to achieve final concentrations of 0.1, 0.5, or 2 mol/L, respectively. Then, 5 mL of each solution was transferred into separate Petri dishes.

Similar steps were followed for glucose and fructose solutions, with adjusted quantities. Specifically, 1.8 g, 9.0 g, or 36.04 g of either D(+)-glucose (CELLPURE^®^ > 98% p.a., water free, Carl Roth GmbH) or D(-)-fructose (> 99.5% p.a., Carl Roth GmbH) was dissolved in 100 mL of the hot (~ 70 °C) 0.3% (w/v) agarose solution to obtain 0.1, 0.5, o 2 mol /L solutions. Again, 5 mL of each solution was dispensed into separate Petri dishes.

All the substrates were allowed to cool to room temperature before being used in the experiments.

*Oviposition Amino Acid-Rich Substrates*. Commercial mixture of 8 essential amino acids (Naturally pure, APOrtha ^®^, see Table S1) was used as source for amino acid-rich substrates. For each experiment, a freshly prepared substrate was used, with the amino acids incorporated into agarose to maintain a consistent softness. A 0.3% (w/v) agarose solution was prepared, and once it cooled to approximately 70 °C, 1.0, 3.5, or 5.0 g of the amino acid mixture was added to 100 mL of agarose solution to obtain 1.0%, 3.5%, or 5.0% (w/v) amino acid solutions, respectively. Then, 5 mL of each solution was added into separate 35 × 10 mm Petri dishes.

All substrates were used for the experiments once the Petri dishes had reached room temperature.

*Oviposition Sugar/Amino Acid-Rich Substrates*. In each experiment, a freshly prepared substrate was used, incorporating sugars and amino acids into agarose to ensure consistent softness. First, 3 g of agarose was dissolved in 1000 mL of distilled water, brought to boiling, and then cooled to about 70 °C. Next, 50 g of the amino acid mixture was added and stirred until fully dissolved, creating a base solution of 5% (w/v) amino acids (8-AA) in 0.3% (w/v) agarose.

To prepare a 0.1 mol/L sucrose solution containing 5% (w/v) 8-AA, 3.42 g of sucrose was added to 100 mL of the base solution. Similarly, a 0.5 mol/L fructose or glucose solution with 5% (w/v) 8-AA was prepared by adding 9.0 g of either fructose or glucose to 100 mL of the base solution. Then, 5 mL of each prepared solution was dispensed into separate Petri dishes.

All substrates were allowed to cool to room temperature before being used in the experiments.

*Hardness of Oviposition Substrates*. A force gauge (Sauter FH-50) was used to measure substrate hardness, following a procedure similar to the Rockwell test (Dai et al. 2009). A metal cone indenter (7 mm deep, 9 mm in diameter) was pressed slowly at a 90° angle into the substrate, which was placed in a plastic cup (1.9 cm high, 1.0 cm in diameter). The device recorded the force (in Newtons). This force was then divided by the square of the cone’s diameter (in millimeters) to obtain a hardness value in N/mm².

### Behavioral Assays

#### Egg Laying Assay

*Four-Choice Assays*. Five virgin female and five virgin male wild-type flies (6 days old) were placed together in a small vial with a plug soaked in 0.1 mol/L sucrose solution for 24 h (“preparation time”). After this period, one mated female was transferred to a plastic cage (10 × 8 × 11 cm) containing four Petri dishes, each filled with 5 mL of different oviposition substrates. The position of the substrates in the cage was randomized.

The oviposition test was conducted from ZT7-ZT8 and lasted 24 h, under conditions of 23 °C, 70% relative humidity, and a 12:12-hour light: dark cycle. The number of eggs laid on each substrate was then counted (Fig. 1A).

A new plastic cage was used for each replicate to avoid the calorie-induced secreted factor (CIF), a droplet expelled by the flies from their gut end after consuming nutritive sugars (Abu et al. 2018).

*Two-Choice Assay*. The following modifications used the same procedures as the four-choice assays; however, only two type of substrates were provided. The flies were exposed under the same conditions as in the previous experiments but had access to only two type of substrates during the 24-hour oviposition test, having two Petri dishes per substrate used. Afterwards, the number of eggs laid was counted (Figure S1A).

*Larval Survival Assay*. One day before setting the assay, a group of 25 females and 25 males flies were put together in a big vial with normal food provided in a Petri dish as oviposition substrate. After 18 h, 100 eggs were taken from the Petri dish, and were equally distributed in 10 Petri dishes, which had the neutral substrate (agarose solution 0.3% w/v). Then, the Petri dishes with the eggs were kept in an incubator with 70% humidity, 25 ºC, and 12 h:12 h light: darknes cycle. Every day, the number of larvae was counted until all of larvae had died. The same steps were followed to study the survival rate of larvae on the different concentrations of sucrose, glucose, fructose, amino acids, and combinations of compounds.

*Quantification and Statistical Analysis*. Statistical analysis tests, sample sizes, and corrections for multiple comparisons are given in the text and figure legends. Statistical tests and data visualization were performed with R (R version 4.4.1 (2024-06-14 ucrt)) and GraphPad Prism (version 10.2.3 (2024-04-21). For the oviposition experiments, only flies that laid more than 5 eggs in the oviposition test were used.

## Electronic Supplementary Material

Below is the link to the electronic supplementary material.

Supplementary Material 1

## Data Availability

No datasets were generated or analysed during the current study.
