# Supplementary material for: How Sugar Quality and Concentration Influence Oviposition Preference in Drosophila Melanogaster
Source: J Chem Ecol. 2025 Jun 20;51(4):67. doi: 10.1007/s10886-025-01620-3 (PMC12181135; doi:10.1007/s10886-025-01620-3)
Supplement: Supplementary file 1 — Supplementary Material 1 [file 10886_2025_1620_MOESM1_ESM.pdf]

How Sugar Quality and Concentration Influence Oviposition Preference in *Drosophila melanogaster*.

Journal of Chemical Ecology

Julio Otárola-Jiménez[1,2] (iD 0000-0003-0479-7793), Richard Spehr[3], Bill S. Hansson [1], Markus Knaden[1,4,*]

[1]*Department of Evolutionary Neuroethology, Max-Planck Institute for Chemical Ecology, Jena, 07745, Germany*

[2]*Chemistry School, University of Costa Rica, San Pedro, San José, 11501-2060, Costa Rica*

[3]*Friedrich Schiller University, Jena, 07743, Germany*

[4]Lead contact

*Correspondence: mknaden@ice.mpg.de (MK)

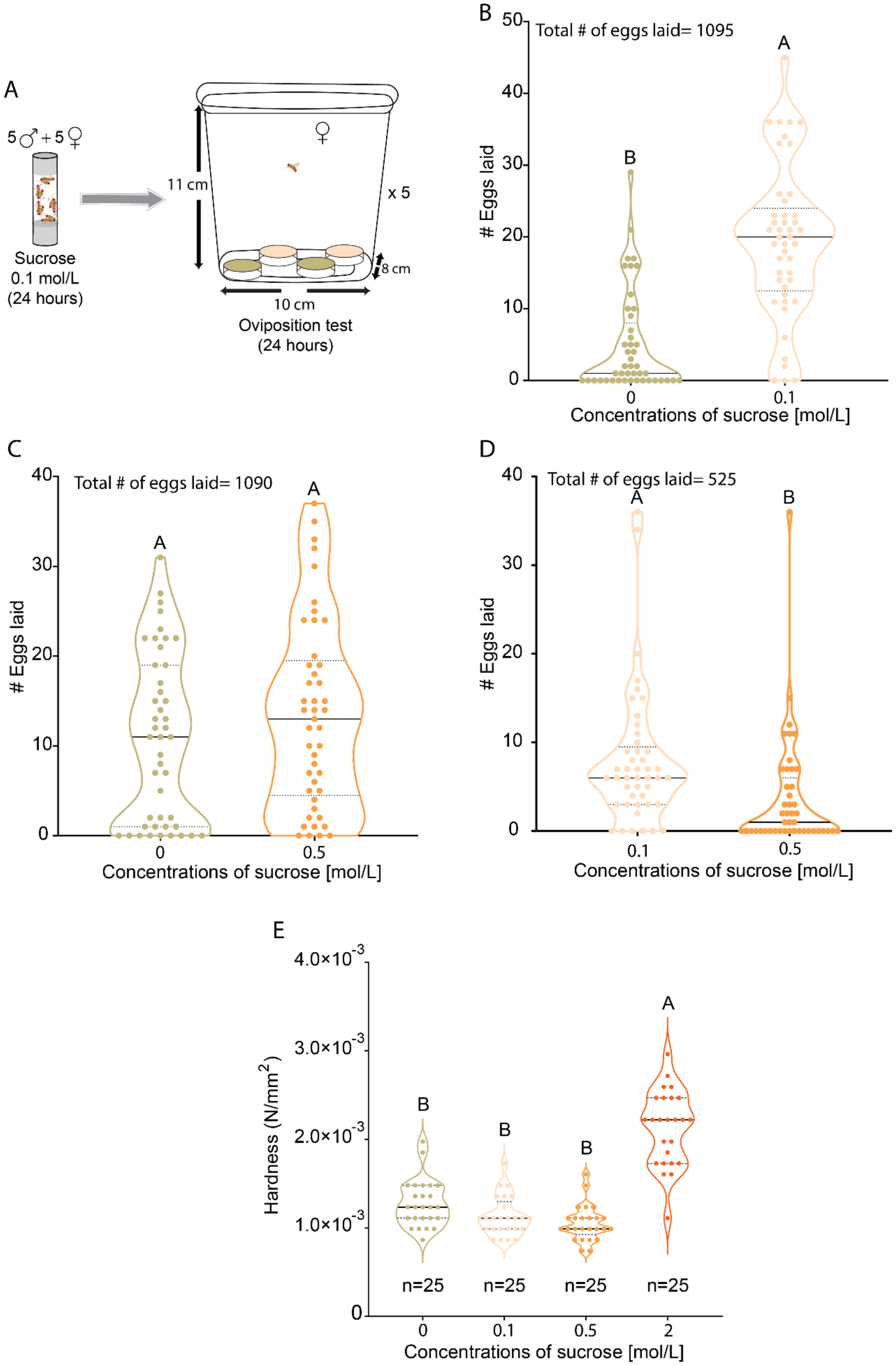

A
5♂+5♀
Sucrose
0.1 mol/L
(24 hours)
11 cm
x 5
8 cm
10 cm
Oviposition test
(24 hours)
B
Total # of eggs laid= 1095
Eggs laid
Concentrations of sucrose [mol/L]
C
Total # of eggs laid= 1090
Eggs laid
Concentrations of sucrose [mol/L]
D
Total # of eggs laid= 525
Eggs laid
Concentrations of sucrose [mol/L]
E
Hardness (N/mm2)
n=25
n=25
n=25
n=25
Concentrations of sucrose [mol/L]

15

Fig.S1 (A) Schematic of the paradigm of two choice assay used to test oviposition preference using sucrose concentrations. (B) Comparison of the number of eggs laid during the oviposition two choice assay between neutral substrate and 0.1 mol/L sucrose. (C) Comparison of the number of eggs laid during the oviposition two choice assay between neutral substrate and 0.5 mol/L sucrose. (D) Comparison of the number of eggs laid during the oviposition two choice assay between 0.1 mol/L and 0.5 mol/L sucrose. (B-D) *Wilcoxon matched-pairs signed rank test* was used (α=0.5, n=45). (E) Comparison of the hardness of the neutral substrate and the different sucrose concentrations. A *Kruskal-Wallis test* followed by *Dunn`s test* for multiple comparison was used (α=0.5, n=25). (B-E) Significant differences (P<0.05) among concentrations are shown with the Compact Letter Display (CLD) system on the top of each violin plot

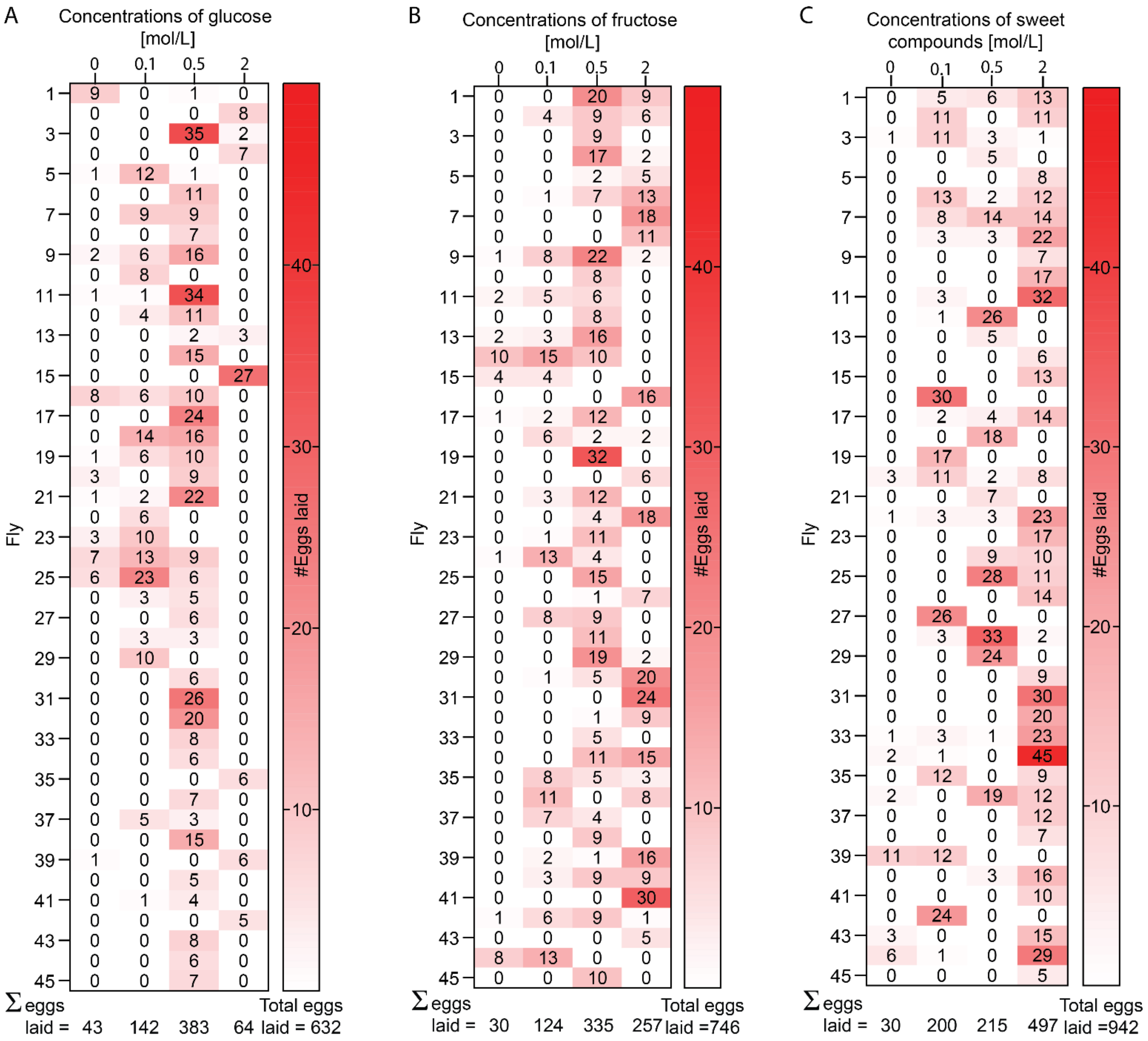

Fig.S2 (A) Head map of the number of eggs of each female laid over 24 hours on different glucose concentrations. (B) Head map of the number of eggs of each female laid over 24 hours on different fructose concentrations. (C) Head map of the number of eggs of each female laid over 24 hours on different sweet compounds at different concentrations

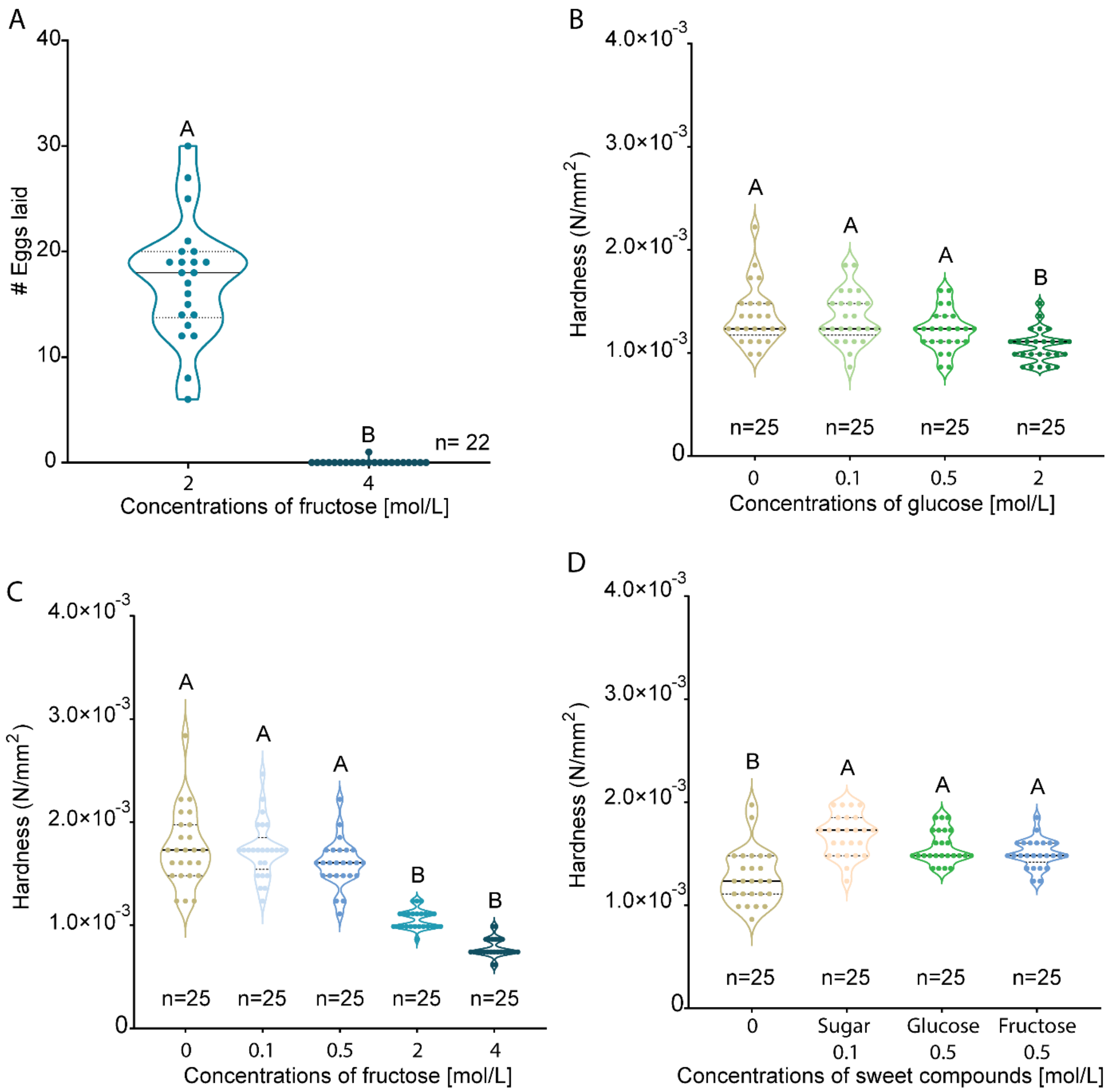

Fig.S3 (A) Oviposition preference between fructose 2 mol/L and 4 mol/L. *Wilcoxon matched-pairs signed rank test* was used (α=0.5, n=22). (B) Comparison of the hardness of the neutral substrate and the different glucose concentrations. (C) Comparison of the hardness of the neutral substrate and the different fructose concentrations. (D) Comparison of the hardness of the neutral substrate and different sweet compounds at different concentrations. (B-D) *Kruskal-Wallis test* followed by *Dunn`s test* for multiple comparison were used (α=0.5, n=25). (A-D) Significant differences (P<0.05) among concentrations are shown with the Compact Letter Display (CLD) system on the top of each violin plot

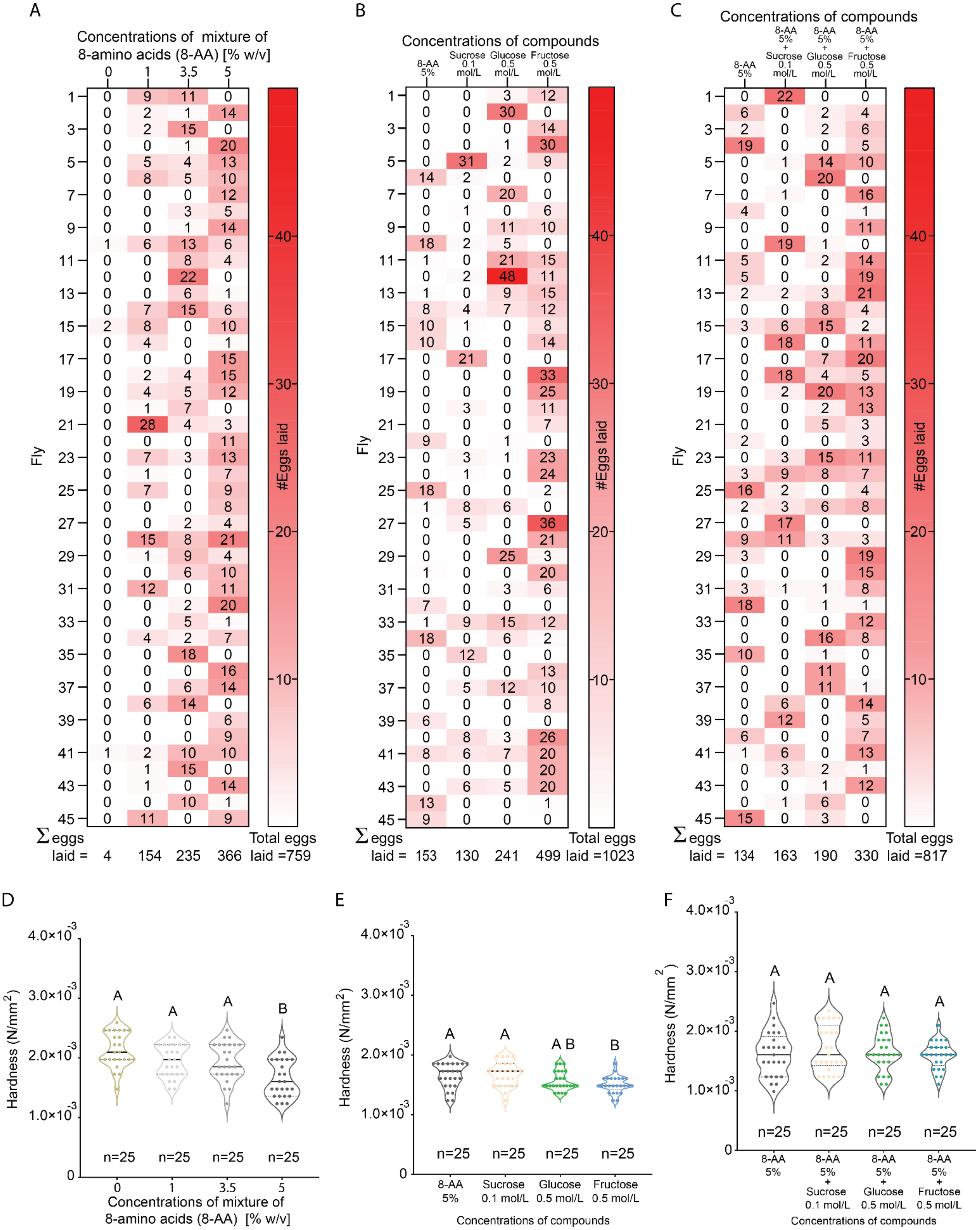
A
Concentrations of mixture of 8-amino acids (8-AA) [% w/v]
0 1 3.5 5
Fly
#Eggs laid
Σ eggs laid = 4 154 235 366
Total eggs laid =759
B
Concentrations of compounds
8-AA 5%
Sucrose 0.1 mol/L
Glucose 0.5 mol/L
Fructose 0.5 mol/L
Σ eggs laid = 153 130 241 499
Total eggs laid =1023
C
Concentrations of compounds
8-AA 5%
8-AA 5% + Sucrose 0.1 mol/L
8-AA 5% + Glucose 0.5 mol/L
8-AA 5% + Fructose 0.5 mol/L
Σ eggs laid = 134 163 190 330
Total eggs laid =817
D
Hardness (N/mm²)
n=25 n=25 n=25 n=25
Concentrations of mixture of 8-amino acids (8-AA) [% w/v]
E
Hardness (N/mm²)
n=25 n=25 n=25 n=25
Concentrations of compounds
F
Hardness (N/mm²)
n=25 n=25 n=25 n=25
Concentrations of compounds

Fig.S4 Head maps of the number of eggs of each female laid over 24 hours on (A) different amino acids mixture concentrations, (B) different chemical compounds, and (C) different combinations of amino acid mixture with sweet compounds. (D) Comparison of the hardness of the neutral substrate and the different amino acids mixture concentrations. (E) Comparison of the hardness of different chemical compounds. (F) Comparison of the hardness of different combinations among amino acid mixture and sweet compounds. (D-F) *Kruskal-Wallis test* followed by *Dunn`s test* for multiple comparison were used (α=0.5, n=25). Significant differences (P<0.05) among concentrations are shown with the Compact Letter Display (CLD) system on the top of each violin plot

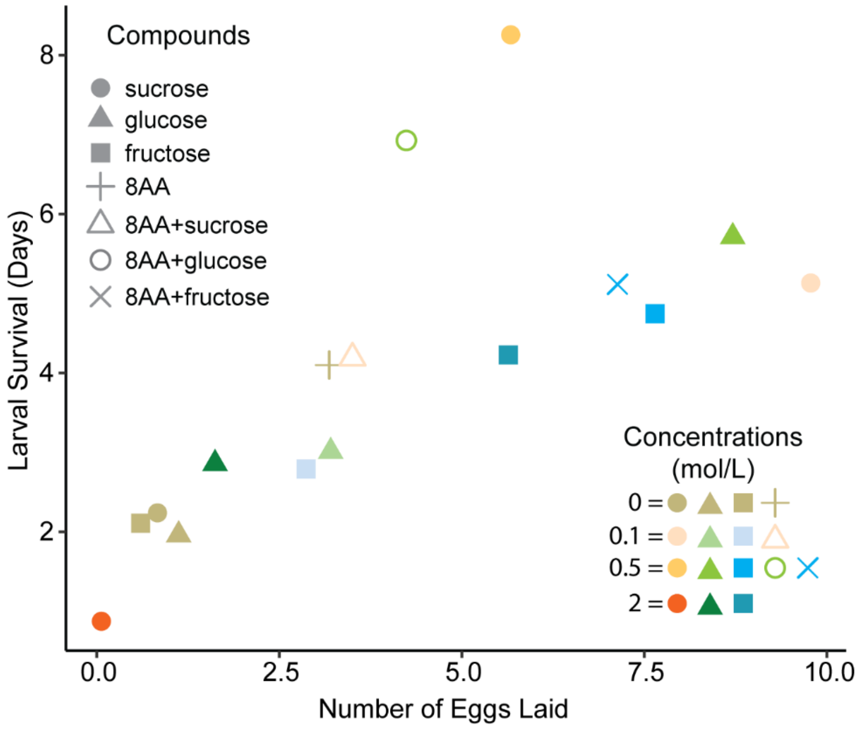

Fig.S5 Scatter plot with the relationship between the number of eggs laid and larval survival (days) across different sugar types, concentrations, and compound combinations. Each symbol represents a treatment condition defined by sugar type, or 8AA (a mixture of 8 amino acids) combined with sugars. Colors indicate sugar concentration (mol/L).

Table S1. Composition of 8 amino acids in the commercial mixture and their physical characteristics related to volatility

Amino acids	% in product	Concentration (mmol/L) in solution 1% w/v	Concentration (mmol/L) in solution 5% w/v	Melting point (°C)[1,2]	Molecular weight (g/mol)[1,2]
**L-Leucine**	19	14.5	72.4	293	131.17
**L-Valine**	16	13.7	68.3	315	117.15
**L-Isoleucine**	14.3	10.9	54.5	285.5 (dec)*	131.17
**L-Lysine**	17.1	11.7	58.5	224.5 (dec)*	146.19
**L-Phenylalanine**	12.4	7.5	37.5	283 (dec)*	165.19
**L-Threonine**	10.6	8.9	44.5	256 (dec)*	119.12
**L-Methionine**	6.7	4.5	22.5	283 (dec)*	149.21
**L-Tryptophan**	3.5	1.7	8.6	290.5 (dec)*	204.22
**Emulsifier (Lecithin)**	0.36	-	-	236-237	758.1

(1) Information from PubChem

(2) Information from the software ChemDraw

dec* means decomposition
